# The Contribution of the Urokinase Plasminogen Activator and the Urokinase Receptor to Pleural and Parenchymal Lung Injury and Repair: A Narrative Review

**DOI:** 10.3390/ijms22031437

**Published:** 2021-02-01

**Authors:** Torry A. Tucker, Steven Idell

**Affiliations:** The Department of Cellular and Molecular Biology, The University of Texas Health Science Center at Tyler, Tyler, TX 75708, USA; torry.tucker@uthct.edu

**Keywords:** urokinase plasminogen activator, urokinase plasminogen activator receptor, fibrinolysis, plasminogen activator inhibitor-1, acute lung injury and repair and pleural injury and pleural organization

## Abstract

Pleural and parenchymal lung injury have long been characterized by acute inflammation and pathologic tissue reorganization, when severe. Although transitional matrix deposition is a normal part of the injury response, unresolved fibrin deposition can lead to pleural loculation and scarification of affected areas. Within this review, we present a brief discussion of the fibrinolytic pathway, its components, and their contribution to injury progression. We review how local derangements of fibrinolysis, resulting from increased coagulation and reduced plasminogen activator activity, promote extravascular fibrin deposition. Further, we describe how pleural mesothelial cells contribute to lung scarring via the acquisition of a profibrotic phenotype. We also discuss soluble uPAR, a recently identified biomarker of pleural injury, and its diagnostic value in the grading of pleural effusions. Finally, we provide an in-depth discussion on the clinical importance of single-chain urokinase plasminogen activator (uPA) for the treatment of loculated pleural collections.

## 1. Introduction

Derangements of pathways of fibrin turnover, including the fibrinolytic system, have long been associated with the pathogenesis of lung and pleural injury and repair [1,2]. In acute and chronic parenchymal lung injury, accelerated coagulation and a fibrinolytic defect favor the formation of extravascular fibrinous transition neomatrices, which can rapidly organize and scar [3,4,5,6]. These events occur in the setting of acute and chronic inflammation, and organization of the fibrinous neomatrices in the alveolar and interstitial lung compartments resemble those associated with microvascular leakage and organization occurring in the context of neoplasia and other inflammatory conditions [7,8,9,10]. In the pleural space, local inflammation likewise leads to increased microvascular permeability, intrapleural egress of coagulation substrates, and inhibitors of fibrinolysis and rapid organization with the formation of fibrinous intrapleural collections or loculae that are capable of impeding pleural drainage [1].

Plasminogen activators (PAs) regulate local fibrinolysis and include tissue plasminogen activator (tPA) and urokinase plasminogen activator (uPA). Both are capable of cleaving plasminogen to generate the active protease plasmin, which, in turn, degrades and remodels fibrin. While tPA is expressed in lung epithelial cells, fibroblasts, and mesothelial cells [2], it is mainly involved in intravascular fibrinolysis [11]. However, tPA has a greater affinity for fibrin than uPA, and its binding to fibrin increases its ability to cleave plasminogen [11]. uPA appears to play a greater role in pericellular proteolysis by virtue of its ability to bind to its receptor, uPA receptor (uPAR) [12]. Plasmin generated by tPA and urokinase plasminogen activator (uPA) reciprocally generates more active two chain forms of the PAs.

In both lung and pleural injury, plasminogen activator inhibitor-1 (PAI-1) appears to play a critical role in outcomes and the process of accelerated organization and scarification. PAI-1 is capable of inhibiting PAs, tPA, and uPA (two chain or active urokinase) within the injured lung and pleural space [1,2]. The activity of these PAs decreases in relation to the severity [13,14] of injury in both compartments [2,15,16]. PAI-1 thereby impedes local fibrinolysis and promotes extravascular fibrin deposition in the injured lung and pleural space [2]. Tissue factor expression by lung epithelial and/or mesothelial cells and lung fibroblasts triggers activation of the coagulation system in these injuries, while fibrinolytic activity is suppressed in resident cells mainly by increased expression of PAI-1 driven by proinflammatory mediators released into the local microenvironment [2,17,18,19,20,21,22,23,24]. These observations and those of other laboratories [13,25,26,27,28,29,30,31,32,33,34,35,36,37,38,39,40,41,42,43,44,45,46,47] support the concept that increments of local coagulation and concurrent decrements of fibrinolysis occur in inflammation and favor the formation and retention of fibrinous extracellular fibrin. Protracted collections of extravascular fibrin can organize with scarification in the injured lung or pleural space [1].

Our purpose in this narrative review is to provide the readers with an overview of the contributions of the urokinase plasminogen activator (uPA) and its receptor; uPAR, in the pathogenesis of lung and pleural injury with particular emphasis on pleural disease. New strategies and approaches to the development of new therapeutic interventions are also reviewed with a review of recent contributions to these fields, building on prior key foundational studies.

## 2. Similar Modes of Regulation Govern the Organization of the Fibrinous Transitional Neomatrix in the Settings of Lung and Pleural Injury

**Figure 1:**
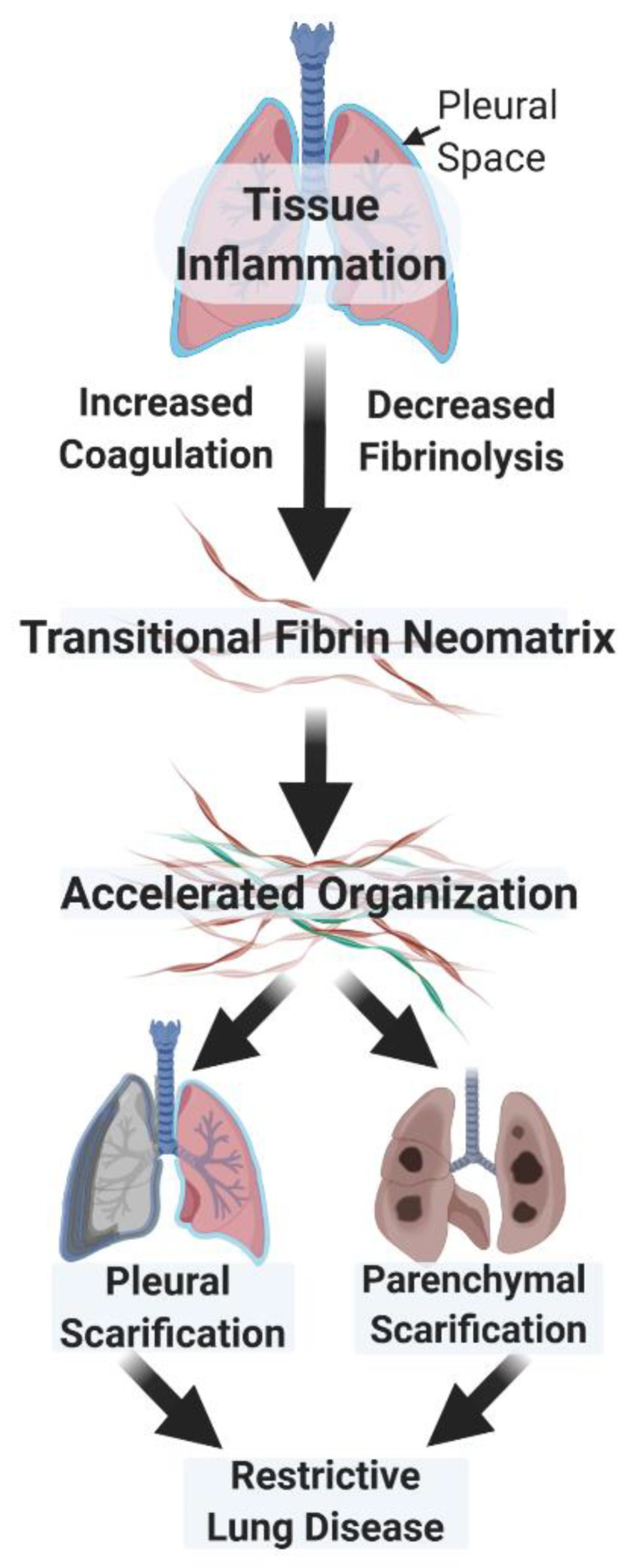
Aberrant fibrin turnover leads to accelerated scarification in the lung or pleural space. The accelerated organization encompasses the invasion of the fibrinous neomatrix by fibroblasts and myofibroblasts, which secrete collagen and initiate fibrotic repair. The neomatrix undergoes continuing remodeling with infiltration by inflammatory cells, including macrophages. Parenchymal lung scarification can be accelerated after acute lung injury or occur more slowly in interstitial lung disease. Pleural scarification can occur with organization, leading to sequestration of pockets of inflammatory pleural fluid; loculation, which can lead to pleural scarification. Pleural fibrosis may also occur after intrapleural bleeding or particulate exposures, such as that due to asbestos. Brown strands indicate fibrin. Green strands indicate collagen intercalated within the fibrinous neomatrix.

Fluid phase and cellular derangements in pathways of fibrin turnover promote fibrin deposition in lung and/or pleural injury. Based on studies of bronchoalveolar lavage and immunohistochemical analyses, uPA is readily detectable in normal lung lining fluids and is the major plasminogen activator represented there [4,5,21,48,49]. While studies of the very small amounts of normal pleural fluid have not, to our knowledge, been conducted, PA activity attributable to uPA and tPA occurs in pleural fluids of patients with congestive heart failure and is generally undetectable after the induction of pleural injury [19]. If unresolved, the inflammatory process suppresses local PA and fibrinolytic activities and perpetuates extravascular fibrin deposition that may rapidly organize over a few days (Figure 1) [3,50]. In the injured lung, early organization promotes accelerated fibroproliferation that can often be detected by lung imaging and can eventuate in lung restriction with long-term, severe morbidity, including respiratory compromise [51]. In the pleural compartment, early organization occurs in the setting of empyema, complicated parapneumonic pleural effusions, hemothoraces, or pleural malignancy and can likewise result in remodeling with loculation or pleurodesis, scarification, pulmonary restriction, and dyspnea [52].

The derangements in pathways of local fibrinolysis have led to the testing of interventions that target fibrin dissolution after lung or pleural injury. While anticoagulant strategies are of conceptual appeal, they have not gained traction for the treatment of patients with pleural or acute lung injury. This may be based largely on clinical trial testing that has failed to show the efficacy in severe sepsis, which is often associated with lung dysfunction [53]. Interestingly, there has been recent consideration of the targeting early pulmonary organization associated with COVID-19 [54]. Whether that approach will be of clinical benefit remains unclear but provocative [55]. Whether lung protection derives from anticoagulants otherwise administered to prevent thrombosis in COVID-19 patients is likewise unclear. On the other hand, in organizing pleural injury associated with loculation and failed drainage, intrapleural fibrinolytic therapy (IPFT) is commonly used and is particularly effective in pediatric patients, as recently reviewed [2].

## 3. The Urokinase/Urokinase Receptor Interaction and Derangements Associated with Lung or Pleural Injury

In the injured lung, pleural space, and virtually all forms of tissue injury, the active two chain form of urokinase or the zymogen single chain urokinase binds to uPAR at the surface of resident cells bearing this Glycosylphosphatidylinositol (GPI)-linked receptor to regulate and localize pericellular proteolysis [56]. When two chain uPA cleaves plasminogen to generate plasmin, plasmin-mediated conversion of single to two chain uPA also occurs to increase the efficiency of pericellular fibrinolysis [57]. Signaling through uPAR can also occur through the binding of uPA or independent of the binding of uPA to uPAR to support the cellular invasion, migration, and cellular viability [56,58]. As uPAR lacks a cytoplasmic domain through which cellular signaling can occur, studies to identify other surface receptors that interact with uPAR are ongoing (Table 1).

Cellular and extravascular derangements of the uPA/uPAR system occur in the setting of acute organizing lung injury. In the injured lung, the profile of alveolar lining fluids assumes more procoagulant and less fibrinolytic potential with increased levels of PAI-1, thereby favoring alveolar fibrin deposition. The alveolar epithelium contributes substantively to these derangements and is capable of regulating its own expression of uPA, uPAR, and PAI-1, which involve unique posttranscriptional mechanisms [59]. The posttranscriptional regulatory mechanisms involve the participation of p53 to, in turn, control the viability of the lung epithelium [59]. The viability of lung epithelial cells is increased when uPA and uPAR are relatively increased. Conversely, apoptosis of these cells is favored by relatively increased expression of PAI-1 with reciprocally decreased uPA and uPAR. In lung fibroblasts harvested from patients with idiopathic pulmonary fibrosis, uPAR expression is likewise increased compared with fibroblasts harvested from the lungs of individuals without lung disease [60]. Regulation of uPAR by lung fibroblasts is, in part, controlled via posttranscriptional regulation, as are pleural mesothelial cells [61].

Preclinical information further supports the critical involvement of the uPA/uPAR system in the pathogenesis of organizing lung injury. Increased uPA expression has been found to mitigate accelerated, fibrosing lung injury induced by bleomycin [41,44,62]. In a related vein, overexpression of PAI-1 aggravated bleomycin-induced lung injury, while PAI-1 deficiency was salutary [63]. Interestingly, uPA or uPAR deficiency did not alter lung hydroxyproline levels in bleomycin-treated mice, but areas of hemorrhage seen in wild-type mice were abridged [42]. On the other hand, uPAR deficiency has been reported to attenuate hypoxia-associated lung injury and reduce lung inflammation, while it likewise impairs inflammation but limits containment of pneumococcal pneumonia with worsened outcomes in mice [64,65].

**Figure 2:**
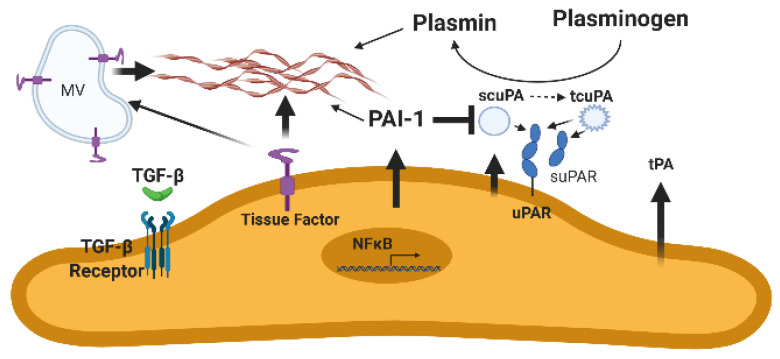
The mesothelium and disordered fibrin turnover. Increased TGF-β expression in response to injury increases plasminogen activator inhibitor-1 (PAI-1) expression. In the aggregate, PAI-1 expression and activity are overexpressed in exudative pleural effusions. This increase in PAI-1 reduces plasminogen conversion to active plasmin by single chain urokinase plasminogen activator (scuPA), two chain urokinase plasminogen activator (tcuPA), and tissue plasminogen activator (tPA). These effects decrease local expression of fibrinolytic activity, which decreases fibrin degradation, leading to aberrant extravascular fibrin deposition. scuPA or the high molecular weight form of tcuPA can bind uPA receptor (uPAR) and be localized to the cell surface or be present unbound in pleural fluids. NFκB is NF kappa B, a signaling mediator that is in particular implicated in the transcriptional regulation of PAI-1 expression by pleural mesothelial cells. MV is microvesicles, which have been shown to contain TF. Brown strands indicate fibrin.

A similar situation exists in the context of pleural injury, where greatly increased pleural fluid PAI-1 and its activity effectively limits local fibrinolysis and predisposes to a rapid, intrapleural organization that can occur over days [16,66,67,68]. In normalcy, the pleural compartment is actually a potential space that expands to form a defined anatomic compartment occupied by inflammatory pleural fluids in pleural infections and other organizing processes [2]. Pleural mesothelial cells express uPA, uPAR, and tPA, which may contribute to fibrinolysis at pleural surfaces. Fibrinolysis is limited by overexpression of PAI-1 in pleural fluids that characterize virtually all forms of pleural injury (Figure 2) [2,23,69]. These pleural collections may undergo rapid organization to loculate, impair pleural drainage and thereby increase morbidity in pleural infection, hemothoraces, or neoplasia. In a murine model of carbon black/bleomycin-induced pleural injury, pleural neomatrix organization was reduced in PAI-1 deficiency but significantly increased by PAI-1 overexpression [70]. Overexpression of PAI-1 has been shown to worsen tetracycline-induced pleural injury [16], and PAI-1-targeted IPFT has been shown to be beneficial [71]. In this model and an empyema model in rabbits, intrapleural administration of PAs has been shown to clear fibrinous pleural collections [2,50,72,73,74,75].

The use of IPFT in clinical practice is likewise predicated on the concept that increments of pleural fluid PA activity can increase intrapleural plasmin generation and fibrinolysis sufficient to improve pleural drainage and clinical outcomes [1,2]. IPFT is generally accepted as effective in pediatric patients and can improve outcomes in adult patients, although adult patient dosing is empiric, and no PA is currently approved for this indication [2,76,77,78].

## 4. The uPA/uPAR System and the Contribution of Mesenchymal Differentiation of Pleural Mesothelial Cells to Pleural Thickening and Scarification

uPAR has been implicated in the pathogenesis of pleural injury and builds upon and extends studies linking uPAR to pleural neoplasia. Interestingly, uPAR has been shown to be involved in the pathogenesis of neoplasia and a target for the development of new therapeutics for several different forms of cancer [79]. In the setting of neoplasia, uPAR expression may be primarily upregulated in the tumor cells themselves or in endothelial or in infiltrating myeloid cells. In malignant pleural mesothelioma cells, uPAR expression correlated with tumor burden, aggressiveness and mortality in mice [58]. Cellular proliferation, migration and invasion were increased in REN malignant mesothelioma cells that have increased uPAR expression. uPAR silencing in these cells decreased cellular indices of aggressiveness, while exposure to uPA and bovine fetal serum enhanced these effects. Conversely, increasing uPAR expression in a less aggressive mesothelioma line significantly increased tumor virulence in vitro and in vivo. The responses suggest the possibility that uPAR-targeted therapeutics now in development may be of value for the treatment of pleural malignant mesothelioma.

Extending this work, uPAR expression by pleural mesothelial cells has been linked to the regulation of pleural remodeling. uPAR internalization was reduced by treatment of human pleural mesothelial cells with TNF-α or IL-1β, mediators which have been implicated in inflammation and the pathogenesis of pleural injury [1,2]. This effect was attributable to decreased expression of the lipoprotein receptor related protein-1; LRP-1, which stabilized uPAR at the cell surface. This stabilization augmented uPA-mediated proteolytic activity at the cell surface, as well as cellular migration [80]. In a murine model of pleural organization induced by carbon black and bleomycin, it was found that PAI-1 deficiency increased pleural thickness and lung restriction likely as a consequence of sustained mesomesenchymal transtion (MesoMT) [70]. The effects involved crosstalk with coagulation proteases and plasmin generation was likewise augmented in pleural lavage of PAI-1-/- mice. Increased plasmin activity was likewise detected in the pleural lavage of mice with empyema induced by intrapleural administration of *Streptoccus pneumoniae* [81], indicating that pleural fibrinolysis is enhanced in pleural injury induced by local instillation of noxious chemicals or bacterial infection.

**Figure 3:**
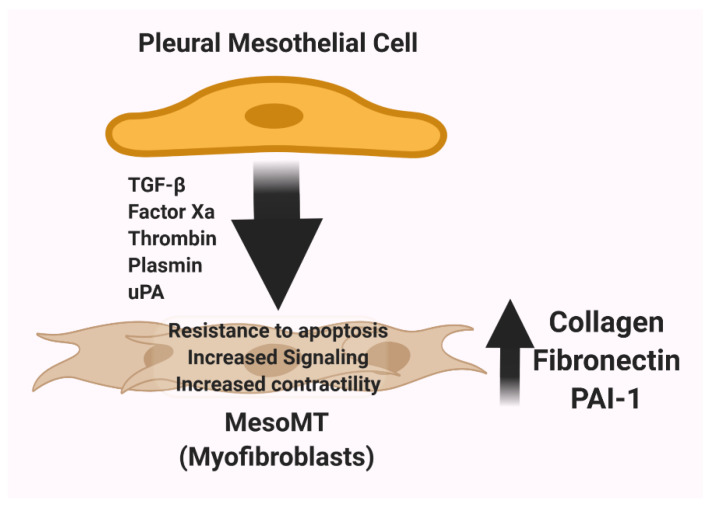
Induction and consequences of MesoMT. Upon stimulation by TGF-β, Factor Xa, thrombin, uPA, or plasmin, pleural mesothelial cells assume a mesenchymal phenotype by undergoing MesoMT. The population of cells undergoing MesoMT expands in the setting of organizing pleural inflammation. Cells undergoing MesoMT elongate compared to unstimulated pleural mesothelial cells and assume the functionality of myofibroblasts and increase expression of neomatrix components including collagen, fibronectin, and PAI-1.

The expansion of the pool of subpleural myofibroblasts contributes to the pleural thickening and neomatrix deposition that characterizes pleural injury and predisposes to the development of pleural fibrosis [69,82,83]. Pleural mesothelial cells have been shown to contribute to that response and undergo a process of mesenchymal phenotypic change called mesomesenchymal transition (MesoMT, Figure 3). The uPA-uPAR system plays a major role in the regulation of this process [1]. In studies to evaluate the impact of coagulation and fibrinolytic proteases on induction of MesoMT, uPA, as well as plasmin, factor Xa and thrombin were found to induce this phenotypic change in human pleural mesothelial cells [70]. Plasmin, thrombin and TGF-b commonly induce MesoMT through phosphatidyl inositol-kinase/AKT/NF kappa (κ) B signaling [84]. The therapeutic targeting of GSK-3β likewise blocked induction of MesoMT and the progression of pleural fibrosis in a novel model of pleural injury [85]. At present, the role of uPA and uPAR in the induction of MesoMT remains to be further elucidated. The effects of proinflammatory cytokines on uPAR stabilization at the surfaces of pleural mesothelial cells via regulation of LRP1 [80], uPA mediated induction of MesoMT [70] and uPA-mediated induction of collagen-1 in these cells suggest a critical role for the uPA/uPAR system in MesoMT and pleural remodeling. Future studies will be needed to define the role of uPAR in the pathogenesis of pleural organization and scarification.

## 5. The Role of Soluble uPAR in Pleural Injury: Biomarker, Effector, or Both?

The structure of uPAR incorporates three domains: DI-III. High affinity binding to its ligand, uPA, primarily involves domain I, while requiring the structural integrity of all three domains [86]. Although a splice variant of the PLAUR gene can generate a soluble uPAR construct [87], soluble uPAR is also generated by cleavage of cell-surface uPAR and occurs in serum and other biologic fluids, including pleural effusions [88,89]. suPAR can be generated by cleavage of the GPI-anchor of uPAR at the cell surface by enzymes such as phospholipase C [90]. Cleavage of a protease sensitive linker region between domains I and domains II-III can also occur via uPA or plasmin among other proteases. Thus, soluble suPAR fragments include domains I-III, I or II-III, as previously reviewed [88]. suPAR, incorporating all domains, retains the ability to bind uPA, via its growth factor domain [91], supporting the concept that this form of suPAR could be a scavenger capable of binding uPA and thereby altering effects of the interaction of uPA with cell surface uPAR such as migration or invasion. 

Interestingly, Higazi and colleagues reported that binding of the proenzyme single chain uPA to suPAR increased its catalytic activity [92]. The authors speculated that scuPA exists in a latent and more active state and that binding of scuPA to suPAR favored the more active conformation, which was more susceptible to inhibition by PAI-1. On the other hand, these observations were disputed by Behrendt and colleagues, who found that the binding of scuPA to suPAR did not accelerate the PA activity of scuPA and in fact had an inhibitory effect on the activation of scuPA to two chain more active uPA by plasmin [93]. The basis for the disparities between these studies remain unclear but are possibly technical. Liberation of domain I from suPAR exposes a chemotactic sequence on the remaining domains (II-III), most of which may be shed from neutrophil membranes, which is a chemotaxin for monocytic cells [94]. Conversely, suPAR domain I has poor affinity for scuPA or uPA and is thereby unable to serve as a scavenger binding either protein [88].

In a recent study, pleural fluid suPAR levels were found to correlate with the requirement for invasive management in parapneumonic pleural effusions [89]. This work extends prior work showing that elevated levels of suPAR occur in a range of biologic fluids in infectious, autoimmune and neoplastic diseases, as previously reviewed [88]. Given the lack of reliable, validated predictors of the need for use of IPFT or surgery for relief of complicated, organizing pleural infections that loculate, Arnold and colleagues sought to determine if pleural fluid or serum suPAR levels predict the need for such management and how suPAR determinations compared with traditional biomarkers such as pleural fluid pH, glucose and lactate dehydrogenase levels. Pleural fluid and serum suPAR levels were determined in 93 subjects with parapneumonic pleural effusions and 47 controls that had either benign transudative or malignant causes of their pleural effusions. A commercially available ELISA assay was used and was able to detect intact suPAR or the domain II-III fragment. The main findings of this important study were that pleural fluid suPAR levels were greater in patients with loculated parapneumonic pleural effusions. Pleural fluid suPAR levels were also superior to pleural fluid pH, glucose, and lactate dehydrogenase (LDH) in combination in terms of predicting the need for IPFT or surgical intervention. Serum suPAR correlated with levels of C-reactive protein, a biomarker of inflammation often used to assess clinical trends in patients with parapneumonic pleural effusions [68]. Pleural fluid suPAR was significantly increased in patients with parapneumonic pleural effusions that were loculated. At a cutoff level of 35 ng/mL, pleural fluid suPAR demonstrated a 100 percent sensitivity, 91 percent specificity for predicting pleural loculation in patients with parapneumonic pleural effusions. Positive and negative likelihood ratios were likewise favorable, and pleural fluid suPAR better predicted loculation than pH. In nine patients with parapneumonic pleural effusions that were initially free-flowing and subsequently loculated, baseline pleural fluid suPAR was elevated to levels equivalent to those found in loculated pleural effusions. Pleural fluid suPAR at the same cutoff was the most accurate biomarker predicting insertion of a chest tube. Lastly, pleural fluid suPAR was superior to the conventional biomarkers in predicting the use of IPFT or surgical intervention and was the only significant baseline parameter that did so. Similar trends were seen in the smaller number of patients with malignancy, in that pleural fluid suPAR was significantly increased in loculated malignant effusions and was non significantly elevated in patients with delayed loculation. The encouraging results suggest that pleural fluid suPAR could improve clinical decision making for the management of organizing pleural infection. The authors properly point out that while pleural fluid suPAR appears to address an unmet clinical need, validation would require a future large, multicenter clinical trial of suPAR-directed clinical management versus current standard management. Whether pleural fluid suPAR can add a precision medicine dimension to such management decisions remains to be proven, but this work underscores the relevance of derangements of the uPA/uPAR system to the field.

## 6. The Use of scuPA for Treatment of Pleural Loculation

For over seventy years, IPFT has been used to treat pleural loculation with failed drainage [2,78]. To date, several agents have been used, now mainly including tPA with or without DNase and urokinase. Many centers have adopted the use of tPA/DNase based on the efficacy demonstrated in adult patients in the MIST-2 clinical trial [95]. Others use tPA alone or two chain urokinase where it is available. Unfortunately, the dosing, administration schedules and agents used for IPFT are all empiric, with no agent currently approved for the indication of treatment of pleural loculation and failed drainage [77]. There are relatively small reports suggesting that two chain uPA (uPA)-based IPFT may be of advantage. uPA-based IPFT was found to be effective in a small randomized, double blinded study of patient with either complicated parapneumonic pleural effusions or empyema [96]. uPA IPFT was also found to be associated with greater efficacy in patients with complicated parapneumonic pleural effusions and less bleeding complications than tPA [97]. In a third small study, uPA was as effective as tPA/DNase for patients requiring IPFT for empyema or parapneumonic pleural effusions but bleeding complications were reduced [98].

Over twenty years ago, we initiated studies in which scuPA, a relatively PAI-1-resistant PA, was used to reverse pleural organization and adhesions in rabbits with tetracycline-induced pleural loculation [50]. Given the similarities of the fibrinolytic system between rabbits and humans, the model was amenable to the testing of human plasminogen activators. We found that scuPA effectively alleviated fibrinous intrapleural collections and adhesions in the model and that single dose treatment was well-tolerated. Single dose administration was as effective as multiple dose scuPA IPFT and generated durable intrapleural PA activity supporting local fibrinolysis [50,72]. In a comparative study, it was found that scuPA was more effective at resolving intrapleural adhesions than any clinically prorated dose of low molecular weight two chain uPA and demonstrated a trend toward better efficacy than tPA [73]. We found that scuPA was processed intrapleurally with formation of bioactive uPA/a2macroglobilin complexes, which appear to contribute to slow release of PA within pleural fluids [99]. In this study, PAI-1 resistant uPA activity was increased in rabbits with tetracycline-induced pleural injury and was related to removal of fibrinous intrapleural collections. PAI-1 resistant enzymatic activity was not found in animals treated with intrapleural low molecular weight uPA or tPA. We identified an equilibrium between an active and relatively inactive form of scuPA, which limited its inactivation by PAI-1 and favored formation of the bioactive uPA/a2macroglobilin complexes.

Based upon these findings and other studies conducted by our group [2], scuPA was manufactured with support by the National Heart Lung and Blood Institute SMARTT (Science Moving towArds Research Translation and Therapy) program, providing sufficient amounts of material for formal toxicology studies needed to enable clinical trial testing. Good Manufacturing Practice (GMP) grade scuPA was also made available for use in clinical trial testing.

## 7. New Strategies to Limit Pleural Organization: Clinical Trial Testing of scuPA for Treatment of Empyema or Complicated Parapneumonic Pleural Effusions

Phase 1 clinical trial testing was next conducted in a multicenter clinical trial performed in Australia Trial Registration: ANZCT ID: ACTRN12616001442493 [68]. In this first-in-kind trial in the field, the safety of scuPA IPFT at daily doses over three days of 50.000–800,000 IU scuPA was tested in patients with complicated parapneumonic pleural effusions or empyema and failed drainage. In all, 14 patients were studied. scuPA was well-tolerated with no bleeding, surgical referrals, or treatment-associated adverse events. In the pleural fluids, scuPA rapidly saturated PAI-1 activity, increased pleural fluid PA and fibrinolytic activities, and generated increased pleural fluid levels of D-dimers. As expected, complexes of uPA/a_2_macroglobilin were generated. No systemic fibrinolysis was detectable, and D-dimer levels remained unchanged from those at baseline in the patients. While this was a safety trial, hints of efficacy were demonstrated in that all but one patient had decreased pleural fluid opacification. At the 800,000 IU dose, pleural sepsis was clinically relieved in both treated patients, and the same effects were observed in two others receiving lower doses of scuPA IPFT.

The favorable Phase 1 trial findings formed the predicate to proceed with Phase 2 efficacy testing, which is now being conducted at multiple sites in the US: ClinicalTrials.gov Identifier: NCT04159831 a phase 2, randomized, placebo-controlled, double-blind, dose-ranging study evaluating LTI-01 (single chain urokinase plasminogen activator, scuPA) in patients with infected, non-draining pleural effusions. The trial design incorporates three doses; 400,000 IU, 800,000 IU, 1,200,000 IU scuPA IPFT, or saline-vehicle placebo, which will be given daily for up to three days. The estimated enrollment is a total of 160 patients or 40 per group. The primary outcome measure is treatment failure due to ongoing pleural sepsis or impaired drainage resulting in surgical referral, and the secondary outcome measure is change in pleural opacity by chest CT scanning at day 4, 1 day after the last IPFT treatment or at the time of treatment failure. The effects of scuPA IPFT on derangements of the fibrinolytic system in pleural fluids will be assessed using the same methods deployed in the Phase 1 trial [68]. This trial is designed to confirm the efficacy of scuPA IPFT in patients with infection-related loculation and failed drainage and to identify an optimal dose of scuPA that can either be used for FDA approval or a follow-up Phase 3 study, as may be required.

## 8. Conclusions

The uPA/uPAR system plays an important role in the pathogenesis of pleural organization through the regulation of local proteolysis, cellular differentiation, and pleural remodeling. In a translational vein, suPAR appears to be a promising new candidate to predict outcomes of pleural injury and to help clinicians to personalize decision making and inform better management of organizing pleural injury. scuPA IPFT is an equally promising translational candidate that is currently in clinical trial evaluation that could offer the first evidence-based, potentially more effective new therapy for patients with nondraining loculated pleural effusions.

## Figures and Tables

**Table 1:** Components of the urokinase plasminogen activator (uPA)/uPA receptor (uPAR) system.

Single Chain uPA; scuPA	A Proenzyme that Can Bind uPAR and Localize PA Activity to the Cell Surface
Two-chain uPA; tcuPA	Conversion of scuPA to this two-chain form generates a much more active PA, can likewise bind uPAR and is mainly involved in pericellular proteolysis.
Tissue type plasminogen activator; tPA	Has a greater affinity for fibrin than tcuPA, relatively more involved in intravascular fibrinolysis, and binding to fibrin increases its PA activity.
Plasminogen activator inhibitor 1; PAI-1	Main PA inhibitor in extravascular fluids in lung and pleural injury, where it can exist in active, cleaved, and inactivated or latent forms. Active PAI-1 can inhibit both tcuPA and tPA.
uPA receptor; uPAR	Multidomain surface glycoprotein responsible for cellular localization of uPA and can be cleaved by uPA. Capable of mediating signaling through interactions with other surface receptors.

## Data Availability

Not Applicable.
